# Microbiota Associated With Cholesteatoma Tissue in Chronic Suppurative Otitis Media

**DOI:** 10.3389/fcimb.2022.746428

**Published:** 2022-04-19

**Authors:** Daniel N. Frank, Jose Pedrito M. Magno, Karen Joyce S. Velasco, Tori C. Bootpetch, Jacob Ephraim D. Salud, Kevin Jer V. David, Aaron L. Miller, Eljohn C. Yee, Heather P. Dulnuan, Richard B. Pyles, Jan Alexeis C. Lacuata, Jeric L. Arbizo, Jennifer M. Kofonow, Beatrice Guce, Kevin Michael D. Mendoza, Charles E. Robertson, Gabriel Martin S. Ilustre, Alessandra Nadine E. Chiong, Shi-Long Lu, Erik A. Tongol, Nicole D. Sacayan, Talitha Karisse L. Yarza, Charlotte M. Chiong, Regie Lyn P. Santos-Cortez

**Affiliations:** ^1^ Division of Infectious Diseases, Department of Medicine, School of Medicine, University of Colorado Anschutz Medical Campus, Aurora, CO, United States; ^2^ Department of Otolaryngology - Head and Neck Surgery, University of the Philippines College of Medicine – Philippine General Hospital, Manila, Philippines; ^3^ Department of Otolaryngology-Head and Neck Surgery, School of Medicine, University of Colorado Anschutz Medical Campus, Aurora, CO, United States; ^4^ Department of Pediatrics, University of Texas Medical Branch, Galveston, TX, United States; ^5^ Philippine National Ear Institute, University of the Philippines Manila – National Institutes of Health, Manila, Philippines; ^6^ Newborn Hearing Screening Reference Center, University of the Philippines Manila – National Institutes of Health, Manila, Philippines; ^7^ Center for Children’s Surgery, Children’s Hospital Colorado, Aurora, CO, United States

**Keywords:** 16S rRNA, cholesteatoma, microbiome, middle ear, otitis media

## Abstract

Otitis media (OM), defined as infection or inflammation of the middle ear (ME), remains a major public health problem worldwide. Cholesteatoma is a non-cancerous, cyst-like lesion in the ME that may be acquired due to chronic OM and cause disabling complications. Surgery is required for treatment, with high rates of recurrence. Current antibiotic treatments have been largely targeted to previous culturable bacteria, which may lead to antibiotic resistance or treatment failures. For this study, our goal was to determine the microbiota of cholesteatoma tissue in comparison with other ME tissues in patients with long-standing chronic OM. ME samples including cholesteatoma, granulation tissue, ME mucosa and discharge were collected from patients undergoing tympanomastoidectomy surgery for chronic OM. Bacteria were profiled by 16S rRNA gene sequencing in 103 ME samples from 53 patients. Respiratory viruses were also screened in 115 specimens from 45 patients. Differences in bacterial profiles (beta-diversity) and the relative abundances of individual taxa were observed between cholesteatoma and ME sample-types. Additionally, patient age was associated with differences in overall microbiota composition while numerous individual taxa were differentially abundant across age quartiles. No viruses were identified in screened ME samples. Biodiversity was moderately lower in cholesteatoma and ME discharge compared to ME mucosal tissues. We also present overall bacterial profiles of ME tissues by sample-type, age, cholesteatoma diagnosis and quinolone use, including prevalent bacterial taxa. Our findings will be useful for fine-tuning treatment protocols for cholesteatoma and chronic OM in settings with limited health care resources.

## Introduction

Otitis media (OM) or middle ear (ME) infection/inflammation remains a major public health problem worldwide, with an estimated 60% of hearing-impaired children under 5 years old having OM as the cause of hearing loss (GBD 2019 Hearing Loss Collaborators, 2021). Risk factors for OM include young age, lack of breastfeeding, allergies, upper respiratory infection, second-hand smoke exposure, low social status, daycare attendance, multiple siblings, and family history (Zhang et al., 2014; Brennan-Jones et al., 2015). OM may persist as recurrent acute (RA)OM or chronic (C)OM, for which treatment includes antibiotics and surgery. In the United States, annual health care use due to office visits, antibiotics, and surgeries for OM in children is estimated to cost >$5 billion (Suaya et al., 2018). Antibiotics are prescribed for 67% of children with OM (Hersh et al., 2016), inappropriately in 10-33% of cases, causing concern for antibiotic resistance (Fleming-Dutra et al., 2016; DeMuri et al., 2017). Globally, age-standardized rates for years lived with disability due to acute OM are still increasing (GBD 2019 Diseases and Injuries Collaborators, 2020). In children, OM can cause not only permanent hearing loss but also impairments in speech perception, auditory processing or phonological awareness while reading, thereby affecting academic performance (O'Niel et al., 2015; Khavarghazalani et al., 2016; Cai and McPherson, 2017; le Clercq et al., 2017; Carroll and Breadmore, 2018). If persisting to adulthood, COM, even with only mild-to-moderate hearing loss, is associated with poorer quality of life and mental health, particularly if revision surgeries were performed (Bakir et al., 2013). After tympanoplasty surgery, the 10-year recurrence rate of COM is 15-26% (Nardone et al., 2012).

Cholesteatoma is a non-cancerous cyst-like lesion in the ME that either develops congenitally (rare) or, more commonly, is acquired in COM. Cholesteatoma was estimated to develop in 10-24% of OM cases (O'Connor et al., 2009; Rosito et al., 2017). In a tertiary hospital setting in Colorado, out of 67,661 children seen over 10 years, 36.5% had OM and 12.0% were diagnosed with COM; of those with COM, 5.1% of children presented with cholesteatoma (unpublished data from COMPASS database). Prevalence is higher in adults: of 1,006 adults with OM, 24.4% had COM; of those with COM, 39.2% had cholesteatoma. These data show that while COM and cholesteatoma are prevalent in lower income countries such as the Philippines (Carrillo et al., 2007; Santos-Cortez et al., 2007), these disease entities are also significant health issues in high-income countries including the United States.

Cholesteatoma has a propensity for insidious growth and erosion of the ossicles or temporal bone that houses neural structures and may lead to disabling complications such as hearing loss, facial nerve palsy, vertigo, or intracranial extension. Surgery is required for treatment, with high rates of recurrence even after >10 years (Kuo et al., 2012; Nardone et al., 2012). Histologically, cholesteatoma is filled by keratin debris and lined by keratinized squamous epithelium (matrix), which is similar to the outer epidermal layer of the tympanic membrane (TM) but with Langerhans cells and basal cells (Lim and Saunders, 1972). Between the matrix and ME mucosa, there is a layer of loose connective tissue (*i.e.*, perimatrix) containing collagen fibers and fibrocytes, typically with inflammation between the perimatrix and ME mucosa (Lim and Saunders, 1972). Under the operating microscope, these tissues can be differentiated well: the cholesteatoma is the encapsulated collection of keratin debris within the ME space, while the hyperplastic, severely inflamed mucosa that is in contact with the cholesteatoma appears as granulation tissue that is distinct from normal-looking but infected ME mucosa (Saunders et al., 2011). Fifty years after its histology was resolved, there is still no consensus on how a cholesteatoma forms, except that the process is probably a hybrid of theories (Maniu et al., 2014; Kuo et al., 2015): [a] desquamated keratin accumulates in a retraction pocket formed by negative ME pressure; [b] squamous epithelium of the TM migrates to the ME; [c] ME mucosa transforms into keratinizing epithelium; [d] keratin-filled microcysts form within the basal layer of the TM and invade subepithelial tissue. Better understanding of cholesteatoma pathology will aid in implementation of standard classifications of lesions to guide management and predict surgical outcomes (James et al., 2019). It was suggested that an exaggerated inflammatory response causes cholesteatoma growth, proliferation, and bony erosion (Maniu et al., 2014; Kuo et al., 2015). Biofilm formation and aberrant gene expression are also associated with cholesteatomas (Chole and Faddis, 2002; Maniu et al., 2014; Kuo et al., 2015; Baschal et al., 2019).

*Pseudomonas aeruginosa* and *Staphylococcus aureus* are the most prevalent bacteria cultured from suppurative COM and cholesteatomatous OM (Ricciardiello et al., 2009). In previous bacterial profiling between different patients, the relative abundances of *Alloiococcus, Haemophilus* and *Clostridiales* were increased in cholesteatomas vs. healthy ME, non-cholesteatomatous OM, or tympanosclerotic plaques (Neeff et al., 2016; Kalcioglu et al., 2018). In another recent study of untreated cholesteatoma, no differences in bacterial or fungal profiles were found between cholesteatoma and ME mucosa (Weiss et al., 2019). In these previous microbiota studies, no biodiversity or relative taxa abundance estimates were significant, whether when comparing by case-control status or by sample-types, and when correcting for multiple testing [e.g. false discovery rate (FDR)] (Neeff et al., 2016; Minami et al., 2017; Kalcioglu et al., 2018; Weiss et al., 2019). On the other hand, in other studies up to 36% of cholesteatomas were reported as positive for human papillomavirus (HPV), a virus that can induce aggressive growth of tissue lesions such as papillomas or cancers (Bergmann et al., 1994; Franz et al., 2007).

In this study, we collected ME samples from Filipino patients undergoing tympanomastoidectomy for COM and submitted them for [1] broad-range bacterial 16S rRNA gene amplification and sequencing and [2] PCR screening for HPV and common viral otopathogens. Our goal was to determine if the microbiota of cholesteatoma tissue is different from other ME samples within the context of long-standing, insufficiently treated suppurative COM.

## Materials And Methods

### Study Design

Prior to initiation, this study was approved by the University of the Philippines Manila Research Ethics Board (UPMREB 2015-238-01) and the Colorado Multiple Institutional Review Board (protocols 16-1525, 16-2673, and 17-1679). Informed consent was obtained from all study participants, including parents of minors. Patients who were diagnosed to have COM with or without cholesteatoma and scheduled for tympanomastoidectomy surgery at the Philippine General Hospital were recruited for study. Preparation for surgery was done according to standard procedures. No antiseptic or antibiotic wash or powder was directly applied to the middle ear before or during surgery. During surgery, different types of ME samples, namely cholesteatoma tissue, ME discharge (obtained by sterile Puritan applicator swab), ME mucosal tissue, and granulation tissue, were collected per patient. All tissues and discharge were collected by the surgeon upon identification under the operating microscope. Each ME sample was placed separately in an Oragene P117 kit (DNA Genotek, Ottawa, Ontario, Canada), which preserved microbial DNA/RNA while shipping to Colorado. Microbial DNA and RNA were isolated from all ME samples using the MasterPure Complete DNA and RNA Purification Kit (Lucigen, Middleton, WI, USA) for DNA samples and the AllPrep DNA/RNA/miRNA Universal Kit (Qiagen, Hilden, Germany) for RNA samples, respectively.

### Microbiota Profiling

Bacterial 16S rRNA genes were amplified from DNA samples using primers specific for the V1V2 region (27FYM 5’-AGAGTTTGATYMTGGCTCAG and 338R 5’-TGCTGCCTCCCGTAGGAGT), as previously described (Santos-Cortez et al., 2018; Bootpetch et al., 2020; Frank et al., 2020). All work was performed in either a biosafety level 2 hood or a HEPA-filtered PCR hood following 15 minutes of ultraviolet irradiation. Three to four negative reagent controls were included in each batch of DNA extraction, PCR, and sequencing to detect contaminants. PCR amplicons were normalized and pooled using a SequalPrep Normalization Plate Kit (Invitrogen/Thermo Fisher Scientific, Waltham, MA, USA) and pools quantified using a Qubit^®^ 2.0 Fluorometer (Invitrogen). Paired-end sequencing was conducted on the Illumina Miseq platform using the 600 cycle v3 kit (San Diego, CA, USA). Illumina Miseq paired-end reads were aligned to human reference genome hg19 with bowtie2 and matching sequences discarded (Langmead and Salzberg, 2012). The remaining non-human paired-end sequences were demultiplexed then assembled using phrap (Ewing and Green, 1998; Ewing et al., 1998). Pairs that did not assemble were discarded. Assembled sequence ends were trimmed over a moving window of 5 nucleotides until average quality was met or exceeded 20. Trimmed sequences with more than 1 ambiguity or shorter than 250 nt were discarded. Potential chimeras identified with Uchime (usearch6.0.203_i86linux32) (Edgar et al., 2011) using the Schloss (Schloss and Westcott, 2011) Silva reference sequences were removed from subsequent analyses. Assembled sequences were aligned and classified with SINA (1.3.0-r23838) using the 418,497 bacterial sequences in Silva 115NR99 as reference configured to yield the Silva taxonomy (Pruesse et al., 2007; Pruesse et al., 2012; Quast et al., 2013). Taxonomic assignment by SINA used the lowest common ancestor approach with default parameters. Operational taxonomic units (OTUs) were produced by binning sequences with identical taxonomic assignments. This process generated a median of 71,960 sequence/sample (IQR: 17391-107146) for 103 ME samples. In contrast, 12 negative control samples yielded a median of 21.5 (IQR: 17-184.3) sequences per control sample.

### Statistical Analysis

The software packages R v4.1.0 (Team, 2019) and Explicet v2.10.5 (Robertson et al., 2013) were used to analyze and visualize data. For microbiome analysis, differences in overall composition (i.e., beta-diversity) were assessed through permutational ANOVA [PERMANOVA (Anderson et al., 2011; Oksanen et al., 2019)] with the Bray-Curtis dissimilarity index. PERMANOVA p-values were inferred through 10^6^ label permutations and FDR-corrected for multiple comparisons (Benjamini and Hochberg, 1995) when multiple pairwise tests were performed. Alpha-diversity indices (i.e., S_obs_, Shannon H, Shannon H/Hmax) were assessed by linear regression modeling; p-values were FDR-adjusted when multiple pairwise tests were performed. PERMANOVA and linear regression were performed on the main outcome (sample-type) both as one-way tests and adjusting for covariates (patient ID, age, sex, cholesteatoma diagnosis, or medications) as noted in the text. Individual taxa differing between treatment groups were identified using the ANOVA-like differential expression (ALDEx2) R package (Fernandes et al., 2013; Fernandes et al., 2014). The distribution of taxa in each sequence library was estimated through 1000 Dirichlet Monte Carlo re-samplings of sequence count data. To account for the compositional nature of microbiome sequence data, datasets were then subjected to a center log-ratio transformation with all features used as the denominator. The aldex.glm module was used to assess between-group differences in taxon relative abundances while adjusting for patient covariates; both nominal p-values and FDR-corrected p-values (Benjamini and Hochberg, 1995) were inferred using this approach, as noted in the text and figures.

### Viral Profiling

To determine whether viruses play a role in cholesteatoma, the ME samples were screened for presence of HPV DNA by qPCR using the PowerUp SYBR Green (Applied Biosystems/Thermo Fisher) reagent and a Bio-Rad instrument (Hercules, CA, USA). We also screened for eight common respiratory viruses that are known to be associated with OM, namely: two DNA viruses – human adenovirus (HAdV) and human bocavirus (HBoV); and six RNA viruses – rhinovirus (RV), enterovirus (EV), respiratory syncytial virus (RSV), coronavirus-229E/NL63/OC43 (hCoV), human metapneumovirus (hMPV), influenza A/B or InfA/InfB [**Table S1**; (Loeffelholz et al., 2011; Nokso-Koivisto et al., 2015)]. Microbial RNA was converted into cDNA using the Superscript IV Reverse Transcriptase (Thermo Fisher) with random hexamer primers. Control plasmids matched to specific primers for each virus were used as positive control. Additionally, beta-actin was used as positive control and was successfully amplified in every sample.

## Results

### Patient Characteristics

Microbial DNA was isolated from 118 specimens from 54 patients enrolled in this study. Out of 118 microbial DNA samples, bacteria were profiled in 103 (87.3%) samples from 53 patients by broad-range 16S rRNA gene PCR amplification and Illumina amplicon sequencing of the V1V2 variable region (**Table 1**). The Goods coverage index was ≥98% for each sample, indicating excellent depth of sequence coverage. The cohort with sequence data was 49% male and had a mean age of 36.8 years old (SD: ± 14.7 years; age range: 15-73 years). Sequence data was available from cholesteatoma tissue (“Chol”; N = 25), granulation tissue (“Gran”; N = 34), ME mucosal tissue (“MEMuc”; N = 17), and ME discharge (“MEDisc”; N = 27; **Table 1**). Almost all patients were diagnosed with suppurative COM, except for two with chronic adhesive OM. Thirty-six patients had cholesteatoma (67.9%, **Table 1**). In addition to hearing loss in all patients, additional complications due to OM were found in 13 (24.5%) patients, including one with brain abscess, six with post-auricular soft tissue abscess, and six patients with dizziness and/or damage to the semicircular canal(s). In one severe case, the patient had acute meningitis, facial paralysis and labyrinthitis. Nine (17%) patients had a previous history of tympanomastoid surgery for COM (i.e. 2-27 years prior to current surgery); of these nine, five patients had cholesteatoma on the same ear while one patient had the previous surgery on the opposite ear.

**Table 1:** Clinical information and collected middle ear (ME) samples from 53 Filipino patients with chronic otitis media.

Variable	Cholesteatoma Tissue	Granulation Tissue	ME Mucosa	ME Discharge	All Samples	All Patients
N	25	34	17	27	103	53
Age (mean ± SD; years)	32.4 ± 16.6	34.1 ± 12.9	35.2 ± 12.6	35.1 ± 17.1	34.1 ± 14.8	36.8 ± 14.7
Sex (%male)	13/25 (52.0)	17/34 (50.0)	9/17 (52.9)	14/27 (51.9)	53/103 (51.5)	26/53 (49.1)
Cholesteatoma (%)	25/25 (100.0)	22/34 (64.7)	11/17 (64.7)	19/27 (70.4)	77/103 (74.8)	36/53 (67.9)
Quinolone (%)	10/22 (45.5)	12/28 (42.9)	7/15 (46.7)	12/24 (50.0)	41/89 (46.1)	19/46 (41.3)
Cephalosporin (%)	18/22 (81.8)	26/28 (92.9)	12/15 (80.0)	20/24 (83.3)	76/89 (85.4)	40/46 (87.0)
Other broad-spectrum antibiotics (%)	4/22 (18.2)	4/28 (14.3)	3/15 (20.0)	4/24 (16.7)	15/89 (16.9)	5/46 (10.9)
Steroid (%)	2/22 (9.1)	1/28 (3.6)	0/15 (0)	2/24 (8.3)	5/89 (5.6)	2/46 (4.3)
*FUT2* variant (%)	1/18 (5.6)	2/24 (8.3)	1/8 (12.5)	1/20 (5.0)	5/70 (7.1)	2/39 (5.1)
*SPINK5* variant (%)	2/17 (11.8)	1/23 (4.3)	1/11 (9.1)	2/21 (9.5)	6/72 (8.3)	2/38 (5.3)

### Microbial Diversity of Middle Ear Specimens

**Figure 1:**
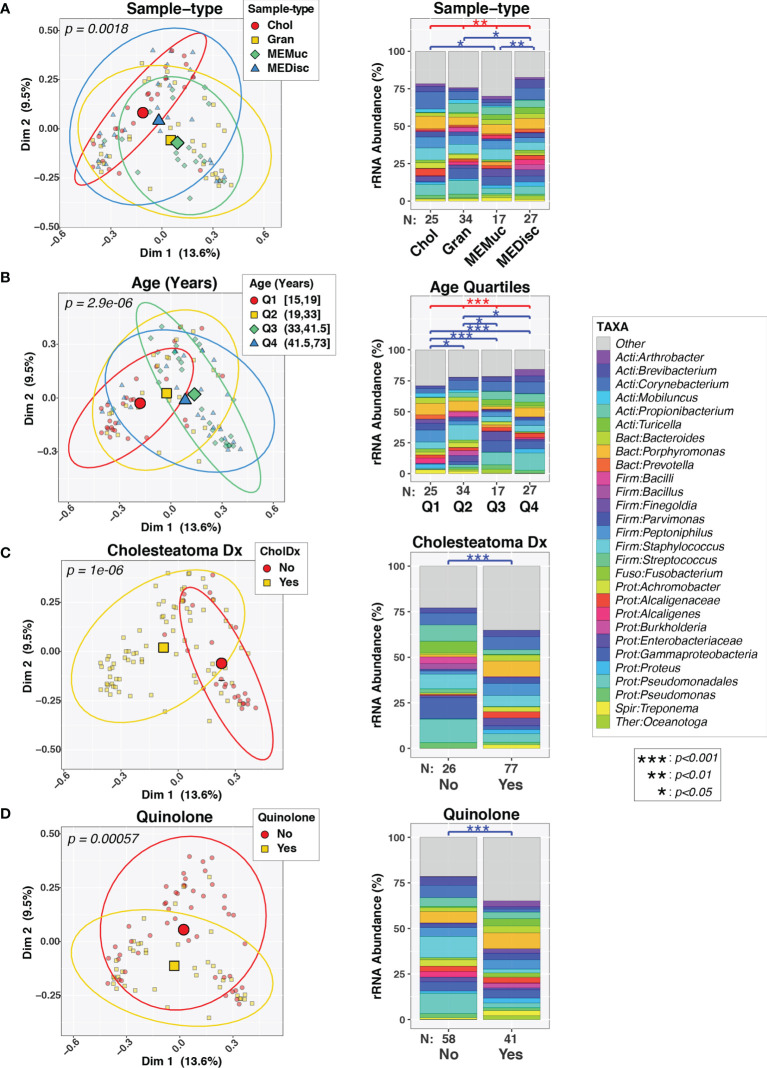
Variability in composition of middle ear microbiota. Pairs of plots show beta-diversity across sample-types **(A)**, age quartiles **(B)**, cholesteatoma diagnosis **(C)**, or quinolone use **(D)**. The left column displays principal coordinates analysis (PCoA) plots of the first two PC axes, color- and symbol-coded by the indicated variables. *Smaller symbols* designate individual subjects while *larger symbols* represent group means along both PC axes. Ellipses designate 90% confidence level for a multivariate t-distribution. The right column displays barcharts summarizing the mean relative abundances of predominant taxa (>2%RA) in each group; rarer taxa are grouped into the “Other” category. Results of PERMANOVA tests are indicated above barcharts. *Red lines/symbols* indicate p-values across all 4 groups. *Blue lines/symbols* indicate FDR-corrected p-values for pairwise comparisons.

Initial univariable PERMANOVA tests found significant associations between overall microbiota composition (i.e., beta-diversity) and sample-type (p=0.006; **Figure 1A**), age (p=2.9e-05; **Figure 1B**), cholesteatoma diagnosis (p=1e-06; **Figure 1C**), quinolone use (p=0.0006; **Figure 1D**) and use of other broad-spectrum antibiotics (p=0.03; data not shown). The effects of these variables on ME microbiota were visualized through both principal component plots (left panels, **Figure 1**) and bar charts (right panels, **Figure 1**), the latter of which also show pairwise differences between different variable levels. In contrast, no differences in beta-diversity were observed in association with biological sex (p=0.20) or cephalosporin use (p=0.37). Only a few patients were positive for [a] use of local steroid as a component of antibiotic otic drops and [b] for *FUT2* or *SPINK5* variants (**Table 1**), which we have previously shown to be determinants of ME microbiota (Santos-Cortez et al., 2018; Frank et al., 2020; Elling et al., 2022); consequently, steroid use, *FUT2* genotype, and *SPINK5* genotype were excluded from all analyses.

**Figure 2:**
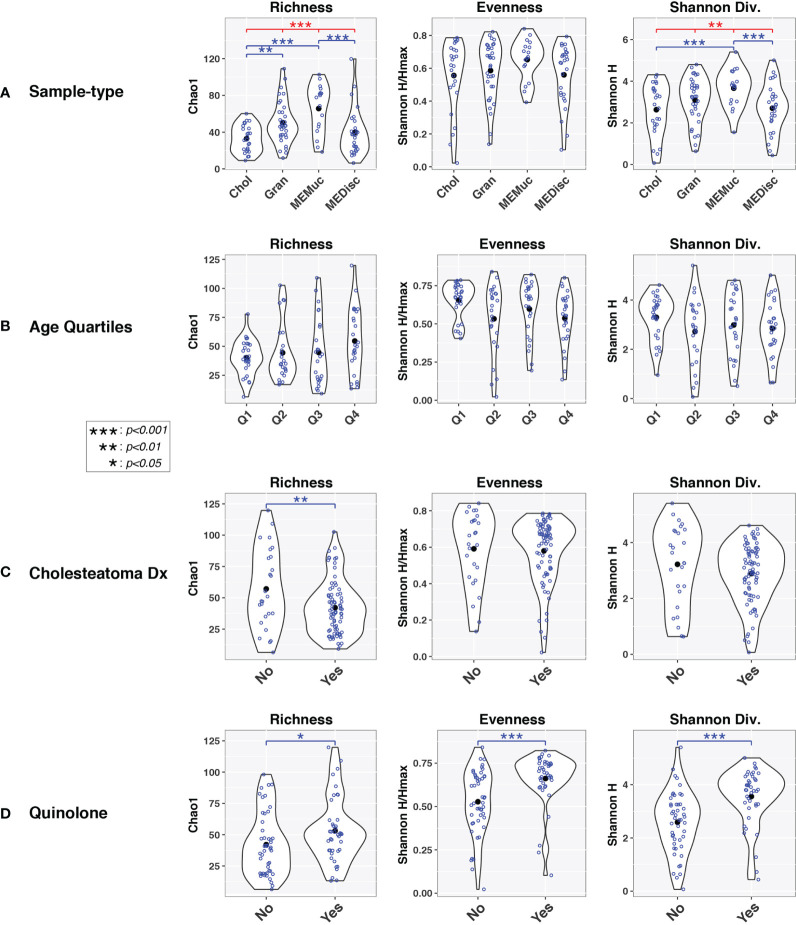
Alpha-diversity indices vary by sample-type and age. *Violin plots* show within-group distributions of values across sample-types **(A)**, age quartiles **(B)**, cholesteatoma diagnosis **(C)**, or quinolone use **(D)**. *Open blue circles* indicate individual samples while *filled black circles* designate group means. Results of linear mixed-effects tests are indicated above violin plots. *Red lines/symbols* indicate p-values across all four groups. *Blue lines/symbols* indicate p-values for pairwise comparisons with false discovery rate correction.

Alpha-diversity indices were also influenced by sample-type, cholesteatoma diagnosis, and quinolone use in univariable analyses (**Figure 2**). Age had little, if any, effect on alpha-diversity. In general, microbial diversity was increased in MEMuc samples compared to Chol and MEDisc samples as measured by richness (Chao1) and Shannon diversity (H) indices, while Gran samples were intermediate between these extremes. Cholesteatoma diagnosis was associated with decreased richness (p=0.006), whereas patients treated with quinolone antibiotics exhibited significantly increased richness (p=0.04), evenness (p=0.0009), and Shannon diversity (p=3.0e-05).

Regardless of the sample-type collected, 16S rRNA gene sequence profiles (**Figure 1**) were dominated by a diverse set of bacteria belonging to the phyla Actinobacteria (e.g., *Corynebacterium*), Bacteroidetes (e.g., *Prevotella*), Firmicutes (e.g., *Bacillus, Streptococcus*), and Proteobacteria (e.g., *Stenotrophomonas*). Of the 157 taxa identified in the set of Chol samples, 18 taxa were observed in ≥75% of the Chol samples (**Figure S1**, **Table S2**), defining a core microbiota for cholesteatomas. Fourteen of these taxa were observed at ≥75% prevalence in all four sample-types (**Figure S1**), while 30 taxa were observed at ≥50% prevalence in all sample-types (data not shown). The highly prevalent, shared taxa consisted of a variety of commensal and potentially pathogenic bacteria from genera such as *Corynebacterium, Propionibacterium, Prevotella, Staphylococcus, Streptococcus, Haemophilus*, and *Pseudomonas.*

**Figure 3:**
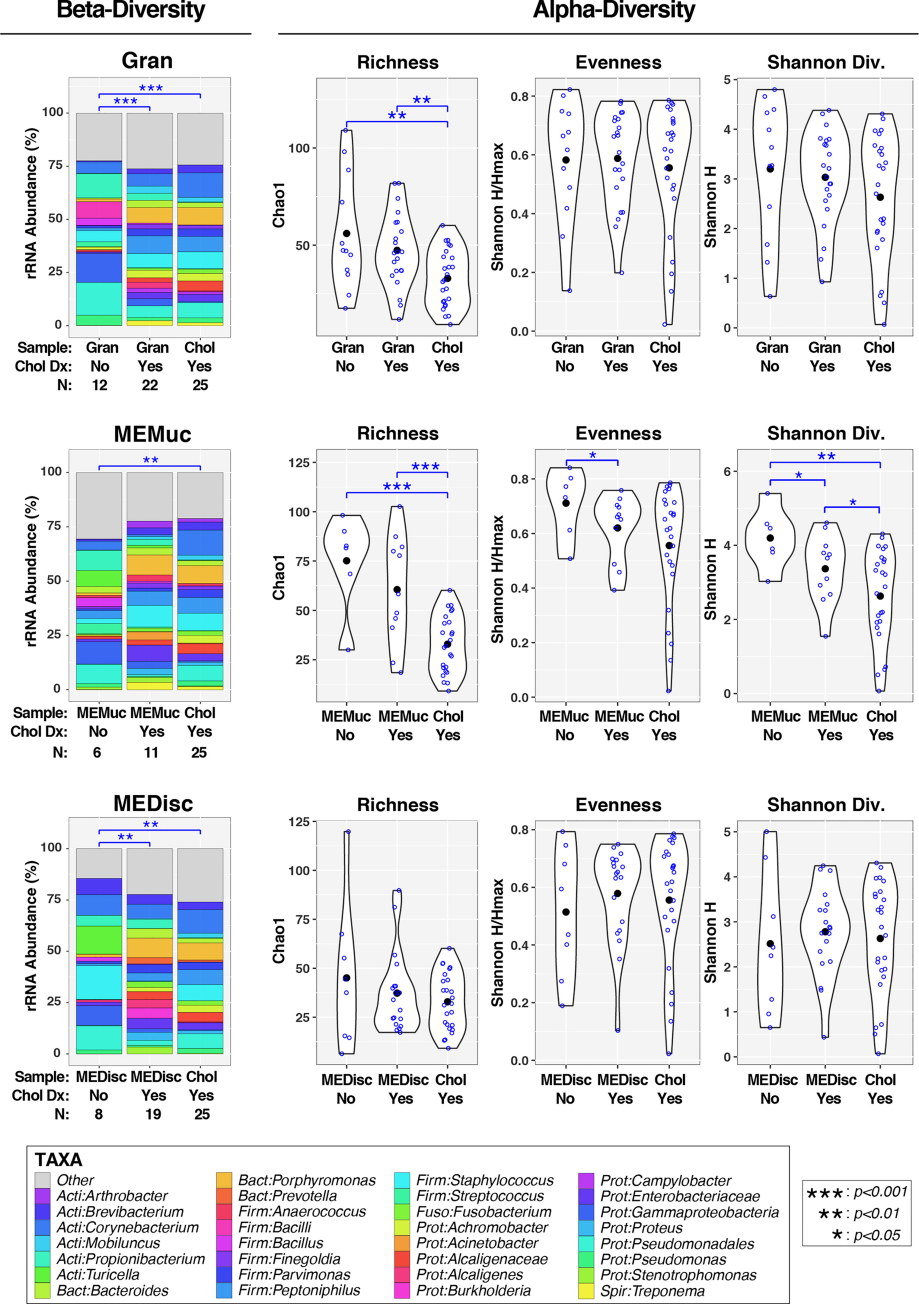
Association of cholesteatoma diagnosis with middle ear microbiota. Each panel compares microbiota for either Gran, MEMuc, or MEDisc samples between patients without and with cholesteatoma diagnosis; for comparison, each panel includes Chol samples from patients with cholesteatoma. The left column (under the heading “Beta-Diversity”) displays barcharts summarizing the mean relative abundances of predominant taxa (>2%RA) in each comparison group; rarer taxa are grouped into the “Other” category. Results of PERMANOVA tests (adjusted for age and quinolone use) are indicated above barcharts. *Blue lines/symbols* indicate FDR-corrected p-values for pairwise comparisons. The right columns (under the heading “Alpha-Diversity”) display violin plots for each comparison group. *Open blue circles* indicate individual samples while *filled black circles* designate group means. Results of linear mixed-effects tests (adjusted for age and quinolone use) are indicated above violin plots. *Blue lines/symbols* indicate p-values for pairwise comparisons with false discovery rate correction.

We next compared the relative abundances of individual taxa across sample-types and age quartiles (**Figure S2**). Consistent with the beta-diversity results, few taxa were differentially abundant between Chol and the other sample-types, even when relatively lax criteria were used to designate features of interest (nominal-p≤0.1, fold-change≥1.5). No taxa had FDR-corrected p-values≤0.1 in pairwise comparisons of sample-types. In contrast, even when using more stringent cutoffs (i.e., FDR-corrected-p≤0.05, fold-change≥1.5), numerous taxa differed in relative abundance between subjects in the first (Q1) and fourth (Q4) age quartiles (**Figure 3**). Younger age (Q1) was associated with elevated levels of diverse Firmicutes, along with *Mycoplasma* and *Prevotella*, while older subjects (Q4) harbored elevated levels of Proteobacteria (*e.g.*, pseudomonads, comamonads) and *Propionibacterium.*

In a multi-variable PERMANOVA model (**Table S3B**), age (p=0.02), cholesteatoma diagnosis (p=3.6e-05), and quinolone use (p=0.0009) remained significant after adjusting for all other variables, while sample-type approached significance (p=0.09). Use of other broad-spectrum antibiotics was no longer significant (p=0.34) in this analysis (data not shown). Based on these findings, the following sections examine the complex interrelationships between cholesteatoma diagnosis, quinolone use, sample-type, and age in influencing ME microbiota. Although sample-type had less apparent effect than the other factors in driving differences between ME microbiotas, we nevertheless stratified analyses by sample-type. This was because the practical goals of this study were to determine 1) whether choice of sample-type influences microbiome results and 2) whether the effects of medications or other factors on ME microbiota differ by sample-type. Indeed, age, cholesteatoma diagnosis, and quinolone use all varied in their associations with ME microbiota across sample-types (**Table S3**).

### Effects of Cholesteatoma on ME Microbiota

We next assessed how cholesteatoma diagnosis affected microbiota in the different ME sample-types. In addition, we sought to determine whether microbiota of cholesteatoma tissue samples (Chol) differed from the other sample-types (Gran, MEMuc, MEDisc) when analyses were stratified by cholesteatoma diagnosis. In PERMANOVA analyses of beta-diversity that adjusted for age and quinolone use (left panels, **Figure 3**), both Gran (p=0.0008) and MEDisc (p=0.002) samples differed significantly between patients with and without cholesteatoma diagnosis, while no differences were observed in MEMuc samples (p=0.17). Similarly, Chol samples (by default from patients with cholesteatoma diagnosis) also differed significantly from Gran (p=9e-06), MEMuc (p=0.002), and MEDisc (p=0.002) samples from patients without cholesteatoma diagnosis. In contrast, Chol samples did not differ significantly from Gran, MEMuc, or MEDisc subjects with cholesteatoma diagnosis.

In analyses of alpha-diversity (middle and right panels, **Figure 3**), subjects with and without cholesteatoma diagnosis exhibited few differences in Gran, MEMuc, or MEDisc samples; only MEMuc samples from cholesteatoma patients had lower evenness (p=0.05) and Shannon Diversity (p=0.03) compared with non-cholesteatoma patients. Regardless of cholesteatoma diagnosis, Chol tissue samples exhibited decreased richness compared to both Gran and MEMuc samples, while Shannon Diversity was also lower in Chol tissues compared with MEMuc.

**Figure 4:**
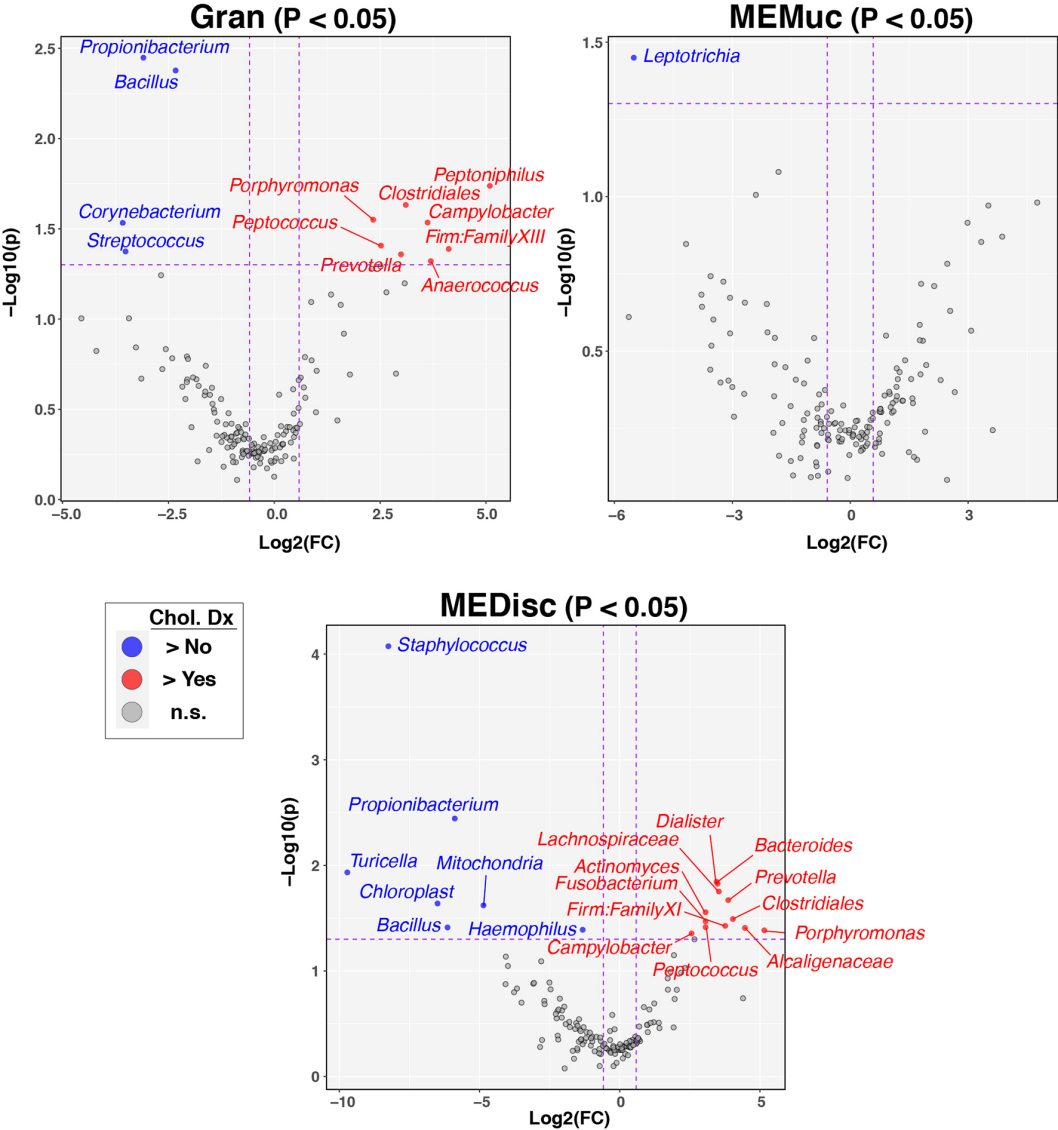
Individual taxa differing between patients with and without cholesteatoma diagnosis. Between-group differences in the relative abundance of individual bacterial taxa were identified using the ANOVA-like differential expression (ALDEx2) test, which considers the compositional nature of microbiota datasets. The three panels show results stratified by sample-type (Gran, MEMuc, or MEDisc). *Vertical dashed lines* indicate fold-change cutoffs≥1.5. *Horizontal dashed lines* show p-value cutoffs for comparisons (nominal p ≤ 0.05). *Blue circles in the upper left quadrants* denote taxa enriched in patients without cholesteatoma diagnosis while *red circles in the upper right quadrants* denote taxa enriched in patients with cholesteatoma diagnosis.

Next, we sought to identify individual taxa differing by cholesteatoma diagnosis and sample-type (**Figures 4**, **S3**). Likely due to the relatively small number of subjects per comparison group, few taxa differed significantly in relative abundance following FDR correction of p-values; consequently, we report exploratory results using less stringent cutoffs (nominal-p≤0.05, fold-change≥1.5). Multiple taxa differed between subjects with and without cholesteatoma diagnosis in both Gran and MEDisc comparisons (**Figure 4**), whereas only one taxon (*Leptotrichia*) met the cutoff criteria for MEMuc. Cholesteatoma diagnosis was associated with increased abundances of the genera *Campylobacter, Peptococcus, Porphyromonas*, and *Prevotella* in both Gran and MEDisc samples. Conversely, the genera *Bacillus* and *Propionibacterium* were enriched in both Gran and MEDisc samples of subjects without cholesteatoma. Finally, among subjects with cholesteatoma diagnosis, only three taxa differed significantly between Chol tissue and MEMuc, while no taxa were differentially abundant between Chol tissues and either Gran or MEDisc (**Figure S3**).

### Effects of Quinolone Antibiotic Use on ME Microbiota

Our initial analyses (**Figure 1**) indicated that quinolone use was significantly and independently associated with altered ME microbiota (beta-diversity) after adjusting for other covariates (age, cholesteatoma diagnosis, sample-type). The effects of quinolone use were observed in subjects both with [p(adjusted)=0.001; adjusted for age and sample-type] and without [p(adjusted)= 0.01; adjusted for age and sample-type] cholesteatoma diagnosis (**Figures S4B**). PCoA analysis (**Figure S4A**) suggested that quinolone use magnified the differences in microbiota observed between subjects with and without cholesteatoma diagnosis. Fewer taxa were differentially abundant in subjects without cholesteatoma (n=3 taxa) than those with cholesteatoma (n=25 taxa), perhaps reflecting the differences in sample size between these groups (**Figures S4C, D**). In cholesteatoma patients, quinolones reduced the abundances of *Corynebacterium, Staphylococcus*, and diverse Proteobacteria (e.g., *Haemophilus, Enterobacter*), with concomitant increases in Firmicutes (e.g., *Peptococcus, Enterococcus, Lachnospiraceae*)

Analyses stratified by sample-type (**Figure S5**, **Table S3**) documented that quinolone use had significant effects in Gran [p(adjusted)=0.055] and MEMuc samples [p(adjusted)=0.02], respectively, adjusted for age and cholesteatoma diagnosis), but not Chol [p(adjusted)=0.13] or MEDisc [p(adjusted)=0.24] samples. PCoA plots (**Figure S5A**) provided further evidence that quinolone use had a larger effect on ME microbiota than sample-type. Finally, despite the significant differences in beta-diversity (**Figure S5B**), few taxa were differentially abundant in association with quinolone use in either Gran (n=5 taxa) or MEMuc (n=2 taxa) samples.

### No Viruses Identified in ME Tissues from Chronic OM Patients

For viral studies, 115 ME samples (23 cholesteatoma, 22 mucosal tissue, 35 granulation tissue, 35 discharge/swabs) from 45 patients with COM were screened for nine viruses. No viral presence was detected in any of the screened ME samples.

## Discussion

In this study, we examined the bacterial and viral microbiotas of cholesteatoma tissue and other ME specimens that were collected from Filipino patients undergoing tympanomastoidectomy surgery for long-standing COM. No viruses were identified in any ME sample, indicating that COM with or without cholesteatoma is primarily a bacterial disease. There was strong overlap in the bacteria found between sample-types whether from the same or across different patients (**Figure S1**). Considering that multiple samples were taken from a limited field at the same ME surgery for each ear, the similarity in community composition is somewhat expected. Nonetheless, we found that the cholesteatoma tissues had moderately less biodiversity than did ME mucosal samples as indicated by richness and Shannon diversity indices (**Figure 2**). Likewise, ME discharge had less alpha-diversity than ME mucosal samples, while granulation tissue had higher richness than cholesteatoma samples (**Figure 2**).

Overall, our results indicate that factors such as quinolone use and cholesteatoma diagnosis had much more substantial effects on ME microbiota composition than did the particular sample-type collected (**Figures 1**, **3**, **S4, S5**). For example, granulation tissues and ME discharge from patients with cholesteatoma diagnosis differed substantially from those with suppurative COM without cholesteatoma (**Figure 3**). Overall, these findings suggest that the microbiota of middle ears with cholesteatoma have a different profile than middle ears with suppurative COM only, which may necessitate a change in approach to treatment.

Differences in biodiversity also were documented across ages of patients with COM (**Figures 1**, **2**), with greater diversity in patients >33 years old compared to teenagers (**Figure 1**; Q1 vs Q3 and Q4). To place this finding within context, the Philippine General Hospital serves a large, indigent population from different regions of the country (Santos-Cortez et al., 2007), many of whom may not have had access to specialists in their locality. Thus, the differences by age bracket may, in fact, be reflective of the duration of ME infection, rather than chronological age *per se*. If the study participants did have some form of antibiotic or surgical treatment prior to sample collection, a large majority is assumed to have received sporadic treatment since the initial infection. By usual protocol, at initial consult, the patients are prescribed antibiotic otic drops (e.g., Ofloxacin, Neomycin-Polymixin B with or without steroids). However due to limited facilities and long waitlists, it may take months from the initial consult before surgery is performed. This situation is common among populations with limited access to otologic health care, particularly in lower-income countries or marginalized groups. Taken together, the differences that we observed across age quartiles are likely to reflect [1] long-standing, untreated or inadequately treated OM, and [2] recent antibiotic use on top of a potentially long history of antibiotic prescription (local or oral) and high likelihood of antibiotic resistance (Xu et al., 2020).

In addition to quinolone, other antibiotics may also have indirectly influenced our findings on ME bacterial profiles. Notably *Pseudomonas*, which is a common target of antibiotic otic drops, had greater relative abundance in older patients (**Figure S2**). This finding may indicate antibiotic resistance and/or extensive formation of treatment-resistant biofilms due to long-standing infection. On the other hand, the most abundant taxa in cholesteatoma tissues were *Corynebacterium*, *Porphyromonas*, and *Staphylococcus*, with 11.5%, 8.3%, and 8.0% mean abundances (**Table S1**, **Figure 1**). *Staphylococcus* is often isolated from COM samples, but may be resistant to penicillin, macrolides or quinolones (Xu et al., 2020).

*Corynebacterium* is also commonly identified in COM but is typically viewed as a commensal, even potentially otoprotective in acute OM (Lappan et al., 2018; Jorissen et al., 2021). In contrast, *Porphyromonas*, *Fusobacterium* and *Campylobacter* (**Figure 1**, **Figure 3**) are less commonly isolated from patients with COM or cholesteatoma (Brook, 1995; Yusuf et al., 2015). *Porphyromonas* and *Fusobacterium* are more common in other anaerobic infections of the head and neck, such as within the oral cavity, oropharynx, and sinuses (Brook, 2011; Yusuf et al., 2015). However, recent 16S rRNA sequencing has identified these bacteria more often in COM cases, indicating the utility of sequencing towards greater understanding of the microbial communities in COM (Santos-Cortez et al., 2016; Johnston et al., 2019). Similar to anaerobic infections of the sinuses and head and neck tissues, the presence of *Porphyromonas* or *Fusobacterium* is likely due to seeding of necrotic or damaged tissue by anaerobic bacterial pathogens after failure of antibiotic treatment (Brook, 2011). These anaerobes may also facilitate biofilm formation (Wang et al., 2018). Identification of these bacteria in cholesteatoma samples that may not be reached by and/or are resistant to the usual otic drops suggest an important opportunity to administer intravenous or oral antibiotics with proper bacterial coverage (e.g., clindamycin, metronidazole, chloramphenicol) immediately after cholesteatoma removal. When the post-surgical ear dressing or packing is removed, instillation of antibiotic drops according to culture and sensitivity test results will help minimize risk of COM recurrence. Local information on antimicrobial susceptibility patterns, which may vary considerably within a few years (del Rosario et al., 1990; Abes et al., 1998; Ayson et al., 2006; Suzuki et al., 2015; Suzuki et al., 2020), will need to be updated regularly and is necessary to efficiently treat COM and cholesteatoma, particularly for the patients described here who lack regular access to health care. Also at the local level, the range of ototopical prescriptions might need to be expanded in order to include antibiotics with proper coverage of target bacteria for post-surgical treatment of cholesteatoma, e.g. chloramphenicol, clindamycin, bacitracin, gramicidin, sulphacetamide, or rifampicin otic drops (van Dongen et al., 2015; Brennan-Jones et al., 2020; de Jong et al., 2020).

There are several limitations of this study. The limited sample size allowed us to identify significant differences in alpha- and beta-diversity but may have been too limited to comprehensively identify differentially abundant taxa, particularly after FDR-correction. The differences in the surgical findings per patient also did not allow us to collect all four sample-types from each patient and reduced the number of samples for paired analyses. Because of restricted access to the ME and to the patients included in this study, all analyses are cross-sectional rather than longitudinal. Lastly, short-read 16S rRNA sequencing limits phylogenetic resolution and, therefore, we took the conservative approach of classifying sequences only to the genus-level. Furthermore, microorganisms such as archaea, fungi, and viruses were not targeted for broad-range sequencing. If feasible, given the low microbial biomasses observed in these specimens, future metagenomic sequence analyses will increase the phylogenetic depth and breadth of these studies. Bacterial species/strain identification will be more helpful in future patients whose ME samples may be submitted for culture and testing of antibiotic sensitivity for the taxa identified in this study.

While having a control population with no history of OM is desirable, we purposefully did not aspire to collect samples from healthy controls, (e.g., from patients undergoing cochlear implantation). There is ongoing debate whether the healthy ME is sterile (Jervis-Bardy et al., 2019; Lee et al., 2021). In our own pilot study of ME swabs from healthy ME of cochlear implant patients with no previous history of OM, no microbial DNA was isolated in 4 out of 5 patients. In one patient, DNA was amplified and submitted to sequencing; upon review of clinical history, this patient had a history of tympanomastoidectomy on the ear opposite to the sampling site, suggesting that the sampled ear was also seeded by previous ME infection, and thus not accurately described as a “healthy control”. Our data therefore support the sterile state of the healthy ME (Jervis-Bardy et al., 2019), precluding any comparison with the microbiota of our patients with COM.

To summarize, in this cohort of patients with long-standing COM, we found that age, cholesteatoma diagnosis, and quinolone use were significantly and independently associated with ME bacterial profiles. Biodiversity was moderately lower in cholesteatoma and ME discharge compared to ME mucosal tissues. Cholesteatoma also had less biodiversity than granulation tissue samples. The findings from this study will be useful in guiding surgical or medical treatment protocols for cholesteatoma and COM, especially in settings with limited health care resources. On a more practical level, these findings indicate that the details of patients’ medical histories and demographic factors are more critical for studies of ME microbiota than the choice or availability of particular types of ME specimens.

## Data Availability Statement

Demultiplexed 16S rRNA paired-end sequence data and associated metadata were deposited in the NCBI Sequence Read Archive under Bioproject ID PRJNA748418.

## Ethics Statement

The study was approved by the University of the Philippines Manila Research Ethics Board (UPMREB 2015-238-01) and the Colorado Multiple Institutional Review Board (protocols 16-1525, 16-2673, and 17-1679). All adult participants and parents of minors provided informed consent.

## Author Contributions

Conceptualization – RS-C. Methodology and resources – DF, JM, KV, JS, KD, AM, EY, HD, RP, JL, JA, BG, KM, CR, GI, AC, S-LL, ET, NS, TY, CC, and RS-C. Formal analysis and investigation – DF, TB, JK, CR, S-LL, and RSC. Original draft preparation – DF, TB, and RS-C. Manuscript editing – all authors. All authors contributed to the article and approved the submitted version.

## Funding

This work was funded by NIH-NIDCD grant R01 DC015004 (to RS-C).

## Conflict of Interest

The authors declare that the research was conducted in the absence of any commercial or financial relationships that could be construed as a potential conflict of interest.

## Publisher’s Note

All claims expressed in this article are solely those of the authors and do not necessarily represent those of their affiliated organizations, or those of the publisher, the editors and the reviewers. Any product that may be evaluated in this article, or claim that may be made by its manufacturer, is not guaranteed or endorsed by the publisher.

## References

[B1] Abes G. T. Jamir J. C. Gloria-Cruz T. Gumban V. M. J. Seredrica G. (1998). Comparative Efficacy and Safety of Ofloxacin and Polymyxin Otic Drops for Chronic Suppurative Otitis Media. Philipp J. Otolaryngol Head Neck Surg. 1998, 129–136.

[B2] Anderson M. J. Crist T. O. Chase J. M. Vellend M. Inouye B. D. Freestone A. L.. (2011). Navigating the Multiple Meanings of Beta Diversity: A Roadmap for the Practicing Ecologist. Ecol. Lett. 14 (1), 19–28. doi: 10.1111/j.1461-0248.2010.01552.x 21070562

[B3] Ayson P. N. Lopez J. E. G. Llanes E. G. D. V. (2006). Chronic Suppurative Otitis Media: Bacteriology and Drug Sensitivity Patterns at the Quirino Memorial Medical Center, (2004-2005): A Preliminary Study. Philipp J. Otolaryngol Head Neck Surg. 21 (1&2), 20–23. doi: 10.32412/pjohns.v21i1-2.823

[B4] Bakir S. Kinis V. Bez Y. Gun R. Yorgancilar E. Ozbay M.. (2013). Mental Health and Quality of Life in Patients With Chronic Otitis Media. Eur. Arch. Otorhinolaryngol 270 (2), 521–526. doi: 10.1007/s00405-012-2031-6 22566178

[B5] Baschal E. E. Larson E. D. Bootpetch Roberts T. C. Pathak S. Frank G. Handley E.. (2019). Identification of Novel Genes and Biological Pathways That Overlap in Infectious and Nonallergic Diseases of the Upper and Lower Airways Using Network Analyses. Front. Genet. 10. doi: 10.3389/fgene.2019.01352 PMC6979043 32010199

[B6] Benjamini Y. Hochberg Y. (1995). Controlling the False Discovery Rate: A Practical and Powerful Approach to Multiple Testing. J. R. Statist Soc Ser. B. 57, 289–300. doi: 10.1111/j.2517-6161.1995.tb02031.x

[B7] Bergmann K. Hoppe F. He Y. Helms J. Muller-Hermelink H. K. Stremlau A.. (1994). Human-Papillomavirus DNA in Cholesteatomas. Int. J. Cancer 59 (4), 463–466. doi: 10.1002/ijc.2910590405 7960213

[B8] Bootpetch T. C. Hafren L. Elling C. L. Baschal E. E. Manichaikul A. W. Pine H. S.. (2020). Multi-Omic Studies on Missense PLG Variants in Families With Otitis Media. Sci. Rep. 10 (1), 15035. doi: 10.1038/s41598-020-70498-w 32929111 PMC7490366

[B9] Brennan-Jones C. G. Head K. Chong L. Y. Burton M. J. Schilder A. G. Bhutta M. F. (2020). Topical Antibiotics for Chronic Suppurative Otitis Media. Cochrane Database Syst. Rev. 1, CD013051. doi: 10.1002/14651858.CD013051.pub2 31896168 PMC6956124

[B10] Brennan-Jones C. G. Whitehouse A. J. Park J. Hegarty M. Jacques A. Eikelboom R. H.. (2015). Prevalence and Risk Factors for Parent-Reported Recurrent Otitis Media During Early Childhood in the Western Australian Pregnancy Cohort (Raine) Study. J. Paediatr. Child Health 51 (4), 403–409. doi: 10.1111/jpc.12741 25303240

[B11] Brook I. (1995). Role of Anaerobic Bacteria in Chronic Otitis Media and Cholesteatoma. Int. J. Pediatr. Otorhinolaryngol 31 (2-3), 153–157. doi: 10.1016/0165-5876(94)01080-h 7782173

[B12] Brook I. (2011). Microbiology of Sinusitis. Proc. Am. Thorac. Soc. 8 (1), 90–100. doi: 10.1513/pats.201006-038RN 21364226

[B13] Cai T. McPherson B. (2017). Hearing Loss in Children With Otitis Media With Effusion: A Systematic Review. Int. J. Audiol 56 (2), 65–76. doi: 10.1080/14992027.2016.1250960 27841699

[B14] Carrillo R. J. Yang N. W. Abes G. T. (2007). Probabilities of Ossicular Discontinuity in Chronic Suppurative Otitis Media Using Pure-Tone Audiometry. Otol. Neurotol. 28 (8), 1034–1037. doi: 10.1097/MAO.0b013e31815882a6 17921908

[B15] Carroll J. M. Breadmore H. L. (2018). Not All Phonological Awareness Deficits Are Created Equal: Evidence From a Comparison Between Children With Otitis Media and Poor Readers. Dev. Sci. 21 (3), e12588. doi: 10.1111/desc.12588 28880490 PMC5947145

[B16] Chole R. A. Faddis B. T. (2002). Evidence for Microbial Biofilms in Cholesteatomas. Arch. Otolaryngol Head Neck Surg. 128 (10), 1129–1133. doi: 10.1001/archotol.128.10.1129 12365883

[B17] de Jong A. Youala M. El Garch F. Simjee S. Rose M. Morrissey I.. (2020). Antimicrobial Susceptibility Monitoring of Canine and Feline Skin and Ear Pathogens Isolated From European Veterinary Clinics: Results of the ComPath Surveillance Programme. Vet. Dermatol. 31 (6), 431–e114. doi: 10.1111/vde.12886 32924232

[B18] del Rosario R. Chiong C. M. Chan A. L. Yap E. C. Jamir J. C. Abes G. T. (1990). Microbial Flora in Chronic Otitis Media: Value of Ear Aspirate Culture Studies. Philipp J. Otolaryngol Head Neck Surg. 1990, 58–66.

[B19] DeMuri G. P. Sterkel A. K. Kubica P. A. Duster M. N. Reed K. D. Wald E. R. (2017). Macrolide and Clindamycin Resistance in Group a Streptococci Isolated From Children With Pharyngitis. Pediatr. Infect. Dis. J. 36 (3), 342–344. doi: 10.1097/INF.0000000000001442 27902646

[B20] Edgar R. C. Haas B. J. Clemente J. C. Quince C. Knight R. (2011). UCHIME Improves Sensitivity and Speed of Chimera Detection. Bioinformatics 27 (16), 2194–2200. doi: 10.1093/bioinformatics/btr381 21700674 PMC3150044

[B21] Elling C. L. Scholes M. A. Streubel S. O. Larson E. D. Wine T. M. Bootpetch T. C.. (2022). The *FUT2* Variant C.461G>A (P.Trp154*) Is Associated With Differentially Expressed Genes and Nasopharyngeal Microbiota Shifts in Patients With Otitis Media. Front. Cell Infect. Microbiol. 11. doi: 10.3389/fcimb.2021.798246 PMC8798324 35096646

[B22] Ewing B. Green P. (1998). Base-Calling of Automated Sequencer Traces Using Phred. II. Error Probabilities. Genome Res. 8 (3), 186–194. 9521922

[B23] Ewing B. Hillier L. Wendl M. C. Green P. (1998). Base-Calling of Automated Sequencer Traces Using Phred. I. Accuracy Assessment. Genome Res. 8 (3), 175–185. doi: 10.1101/gr.8.3.175 9521921

[B24] Fernandes A. D. Macklaim J. M. Linn T. G. Reid G. Gloor G. B. (2013). ANOVA-Like Differential Expression (ALDEx) Analysis for Mixed Population RNA-Seq. PloS One 8 (7), e67019. doi: 10.1371/journal.pone.0067019 23843979 PMC3699591

[B25] Fernandes A. D. Reid J. N. Macklaim J. M. McMurrough T. A. Edgell D. R. Gloor G. B. (2014). Unifying the Analysis of High-Throughput Sequencing Datasets: Characterizing RNA-Seq, 16S rRNA Gene Sequencing and Selective Growth Experiments by Compositional Data Analysis. Microbiome 2, 15. doi: 10.1186/2049-2618-2-15 24910773 PMC4030730

[B26] Fleming-Dutra K. E. Hersh A. L. Shapiro D. J. Bartoces M. Enns E. A. File T. M. Jr.. (2016). Prevalence of Inappropriate Antibiotic Prescriptions Among US Ambulatory Care Visits 2010-2011. JAMA 315 (17), 1864–1873. doi: 10.1001/jama.2016.4151 27139059

[B27] Frank D. N. Giese A. P. J. Hafren L. Bootpetch T. C. Yarza T. K. L. Steritz M. J.. (2020). Otitis Media Susceptibility and Shifts in the Head and Neck Microbiome Due to SPINK5 Variants. J. Med. Genet. 58 (7), 442–452. doi: 10.1136/jmedgenet-2020-106844 32709676 PMC8218788

[B28] Franz P. Teschendorf M. Wohlschlager J. Fischer M. (2007). Prevalence of Human Papillomavirus DNA in Cholesteatomas. ORL J. Otorhinolaryngol Relat. Spec 69 (4), 251–255. doi: 10.1159/000101547 17409785

[B29] GBD 2019 Diseases and Injuries Collaborators (2020). Global Burden of 369 Diseases and Injuries in 204 Countries and Territories 1990-2019: A Systematic Analysis for the Global Burden of Disease Study 2019. Lancet 396 (10258), 1204–1222. doi: 10.1016/S0140-6736(20)30925-9 33069326 PMC7567026

[B30] GBD 2019 Hearing Loss Collaborators (2021). Hearing Loss Prevalence and Years Lived With Disability 1990-2019: Findings From the Global Burden of Disease Study 2019. Lancet 397 (10278), 996–1009. doi: 10.1016/S0140-6736(21)00516-X 33714390 PMC7960691

[B31] Hersh A. L. Fleming-Dutra K. E. Shapiro D. J. Hyun D. Y. Hicks L. A. Outpatient Antibiotic Use Target-Setting W. (2016). Frequency of First-Line Antibiotic Selection Among US Ambulatory Care Visits for Otitis Media, Sinusitis, and Pharyngitis. JAMA Intern. Med. 176 (12), 1870–1872. doi: 10.1001/jamainternmed.2016.6625 27775770 PMC6364667

[B32] James A. L. Tono T. Cohen M. S. Iyer A. Cooke L. Morita Y.. (2019). International Collaborative Assessment of the Validity of the EAONO-JOS Cholesteatoma Staging System. Otol. Neurotol. 40 (5), 630–637. doi: 10.1097/MAO.0000000000002168 31083088

[B33] Jervis-Bardy J. Leong L. E. X. Papanicolas L. E. Ivey K. L. Chawla S. Woods C. M.. (2019). Examining the Evidence for an Adult Healthy Middle Ear Microbiome. mSphere 4 (5), e00456–19. doi: 10.1128/mSphere.00456-19 31484741 PMC6731531

[B34] Johnston J. Hoggard M. Biswas K. Astudillo-Garcia C. Radcliff F. J. Mahadevan M.. (2019). Pathogen Reservoir Hypothesis Investigated by Analyses of the Adenotonsillar and Middle Ear Microbiota. Int. J. Pediatr. Otorhinolaryngol 118, 103–109. doi: 10.1016/j.ijporl.2018.12.030 30599284

[B35] Jorissen J. van den Broek M. F. L. De Boeck I. Van Beeck W. Wittouck S. Boudewyns A.. (2021). Case-Control Microbiome Study of Chronic Otitis Media With Effusion in Children Points at Streptococcus Salivarius as a Pathobiont-Inhibiting Species. mSystems 6 (2), e0056–21. doi: 10.1128/mSystems.00056-21 PMC8546964 33879499

[B36] Kalcioglu M. T. Guldemir D. Unaldi O. Egilmez O. K. Celebi B. Durmaz R. (2018). Metagenomics Analysis of Bacterial Population of Tympanosclerotic Plaques and Cholesteatomas. Otolaryngol Head Neck Surg. 159 (4), 724–732. doi: 10.1177/0194599818772039 29688828

[B37] Khavarghazalani B. Farahani F. Emadi M. Hosseni Dastgerdi Z. (2016). Auditory Processing Abilities in Children With Chronic Otitis Media With Effusion. Acta Otolaryngol 136 (5), 456–459. doi: 10.3109/00016489.2015.1129552 26881324

[B38] Kuo C. L. Shiao A. S. Liao W. H. Ho C. Y. Lien C. F. (2012). How Long Is Long Enough to Follow Up Children After Cholesteatoma Surgery? A 29-Year Study. Laryngoscope 122 (11), 2568–2573. doi: 10.1002/lary.23510 23108885

[B39] Kuo C. L. Shiao A. S. Yung M. Sakagami M. Sudhoff H. Wang C. H.. (2015). Updates and Knowledge Gaps in Cholesteatoma Research. BioMed. Res. Int. 2015, 854024. doi: 10.1155/2015/854024 25866816 PMC4381684

[B40] Langmead B. Salzberg S. L. (2012). Fast Gapped-Read Alignment With Bowtie 2. Nat. Methods 9 (4), 357–359. doi: 10.1038/nmeth.1923 22388286 PMC3322381

[B41] Lappan R. Imbrogno K. Sikazwe C. Anderson D. Mok D. Coates H.. (2018). A Microbiome Case-Control Study of Recurrent Acute Otitis Media Identified Potentially Protective Bacterial Genera. BMC Microbiol. 18 (1), 13. doi: 10.1186/s12866-018-1154-3 29458340 PMC5819196

[B42] le Clercq C. M. P. van Ingen G. Ruytjens L. Goedegebure A. Moll H. A. Raat H.. (2017). Prevalence of Hearing Loss Among Children 9 to 11 Years Old: The Generation R Study. JAMA Otolaryngol Head Neck Surg. 143 (9), 928–934. doi: 10.1001/jamaoto.2017.1068 28750130 PMC5710286

[B43] Lee J. Y. Jacob K. M. Kashefi K. Reguera G. (2021). Oral Seeding and Niche-Adaptation of Middle Ear Biofilms in Health. Biofilm 3, 100041. doi: 10.1016/j.bioflm.2020.100041 33665609 PMC7822943

[B44] Lim D. J. Saunders W. H. (1972). Acquired Cholesteatoma: Light and Electron Microscopic Observations. Ann. Otol. Rhinol. Laryngol 81 (1), 1–11. doi: 10.1177/000348947208100102 5009814

[B45] Loeffelholz M. J. Pong D. L. Pyles R. B. Xiong Y. Miller A. L. Bufton K. K.. (2011). Comparison of the FilmArray Respiratory Panel and Prodesse Real-Time PCR Assays for Detection of Respiratory Pathogens. J. Clin. Microbiol. 49 (12), 4083–4088. doi: 10.1128/JCM.05010-11 21998418 PMC3232999

[B46] Maniu A. Harabagiu O. Perde Schrepler M. Catana A. Fanuta B. Mogoanta C. A. (2014). Molecular Biology of Cholesteatoma. Rom J. Morphol Embryol 55 (1), 7–13. 24715159

[B47] Minami S. B. Mutai H. Suzuki T. Horii A. Oishi N. Wasano K.. (2017). Microbiomes of the Normal Middle Ear and Ears With Chronic Otitis Media. Laryngoscope 127 (10), E371–E377. doi: 10.1002/lary.26579 28397271

[B48] Nardone M. Sommerville R. Bowman J. Danesi G. (2012). Myringoplasty in Simple Chronic Otitis Media: Critical Analysis of Long-Term Results in a 1,000-Adult Patient Series. Otol. Neurotol. 33 (1), 48–53. doi: 10.1097/MAO.0b013e31823dbc26 22143300

[B49] Neeff M. Biswas K. Hoggard M. Taylor M. W. Douglas R. (2016). Molecular Microbiological Profile of Chronic Suppurative Otitis Media. J. Clin. Microbiol. 54 (10), 2538–2546. doi: 10.1128/JCM.01068-16 27487953 PMC5035421

[B50] Nokso-Koivisto J. Marom T. Chonmaitree T. (2015). Importance of Viruses in Acute Otitis Media. Curr. Opin. Pediatr. 27 (1), 110–115. doi: 10.1097/MOP.0000000000000184 25514574 PMC4383320

[B51] O'Connor T. E. Perry C. F. Lannigan F. J. (2009). Complications of Otitis Media in Indigenous and Non-Indigenous Children. Med. J. Aust. 191 (S9), S60–S64. doi: 10.5694/j.1326-5377.2009.tb02929.x 19883359

[B52] O'Niel M. B. Cassidy L. D. Link T. R. Kerschner J. E. (2015). Tracking Tympanostomy Tube Outcomes in Pediatric Patients With Otitis Media Using an Electronic Database. Int. J. Pediatr. Otorhinolaryngol 79 (8), 1275–1278. doi: 10.1016/j.ijporl.2015.05.029 26115935

[B53] Oksanen J. Blanchet G. Friendly M. Kindt R. Legendre P. McGlinn D.. (2019) Vegan: Community Ecology Package. R Package Version 2.5-7. Available at: http://vegan.r-forge.r-project.org.

[B54] Pruesse E. Peplies J. Glockner F. O. (2012). SINA: Accurate High-Throughput Multiple Sequence Alignment of Ribosomal RNA Genes. Bioinformatics 28 (14), 1823–1829. doi: 10.1093/bioinformatics/bts252 22556368 PMC3389763

[B55] Pruesse E. Quast C. Knittel K. Fuchs B. Ludwig W. Peplies J.. (2007). SILVA: A Comprehensive Online Resource for Quality Checked and Aligned Ribosomal RNA Sequence Data Compatible With ARB. Nucleic Acids Res. 35 (21), 7188–7196. doi: 10.1093/nar/gkm864 17947321 PMC2175337

[B56] Quast C. Pruesse E. Yilmaz P. Gerken J. Schweer T. Yarza P.. (2013). The SILVA Ribosomal RNA Gene Database Project: Improved Data Processing and Web-Based Tools. Nucleic Acids Res. 41 (Database issue), D590–D596. doi: 10.1093/nar/gks1219 23193283 PMC3531112

[B57] Ricciardiello F. Cavaliere M. Mesolella M. Iengo M. (2009). Notes on the Microbiology of Cholesteatoma: Clinical Findings and Treatment. Acta Otorhinolaryngol Ital 29 (4), 197–202. 20161877 PMC2816367

[B58] Robertson C. E. Harris J. K. Wagner B. D. Granger D. Browne K. Tatem B.. (2013). Explicet: Graphical User Interface Software for Metadata-Driven Management, Analysis and Visualization of Microbiome Data. Bioinformatics 29 (23), 3100–3101. doi: 10.1093/bioinformatics/btt526 24021386 PMC3834795

[B59] Rosito L. P. da Silva M. N. Selaimen F. A. Jung Y. P. Pauletti M. G. Jung L. P.. (2017). Characteristics of 419 Patients With Acquired Middle Ear Cholesteatoma. Braz. J. Otorhinolaryngol 83 (2), 126–131. doi: 10.1016/j.bjorl.2016.02.013 27236633 PMC9442758

[B60] Santos-Cortez R. L. P. Chiong C. M. Frank D. N. Ryan A. F. Giese A. P. J. Bootpetch Roberts T.. (2018). FUT2 Variants Confer Susceptibility to Familial Otitis Media. Am. J. Hum. Genet. 103 (5), 679–690. doi: 10.1016/j.ajhg.2018.09.010 30401457 PMC6217759

[B61] Santos-Cortez R. L. P. Chiong C. M. San Agustin M. L. M. Elgar C. M. C. Gimena G. L. M. Ibrahim S. C.. (2007). The Philippine National Ear Institute: Patient and Audiologic Profiles. Philipp J. Otolaryngol Head Neck Surg. 22, 12–18. doi: 10.32412/pjohns.v22i1-2.789

[B62] Santos-Cortez R. L. Hutchinson D. S. Ajami N. J. Reyes-Quintos M. R. Tantoco M. L. Labra P. J.. (2016). Middle Ear Microbiome Differences in Indigenous Filipinos With Chronic Otitis Media Due to a Duplication in the A2ML1 Gene. Infect. Dis. Poverty 5 (1), 97. doi: 10.1186/s40249-016-0189-7 27799062 PMC5088646

[B63] Saunders J. Murray M. Alleman A. (2011). Biofilms in Chronic Suppurative Otitis Media and Cholesteatoma: Scanning Electron Microscopy Findings. Am. J. Otolaryngol 32 (1), 32–37. doi: 10.1016/j.amjoto.2009.09.010 20036033

[B64] Schloss P. D. Westcott S. L. (2011). Assessing and Improving Methods Used in Operational Taxonomic Unit-Based Approaches for 16S rRNA Gene Sequence Analysis. Appl. Environ. Microbiol. 77 (10), 3219–3226. doi: 10.1128/AEM.02810-10 21421784 PMC3126452

[B65] Suaya J. A. Gessner B. D. Fung S. Vuocolo S. Scaife J. Swerdlow D. L.. (2018). Acute Otitis Media, Antimicrobial Prescriptions, and Medical Expenses Among Children in the United States During 2011-2016. Vaccine 36 (49), 7479–7486. doi: 10.1016/j.vaccine.2018.10.060 30385056

[B66] Suzuki K. Kurono Y. Ikeda K. Hotomi M. Yano H. Watanabe A.. (2020). The Seventh Nationwide Surveillance of Six Otorhinolaryngological Infectious Diseases and the Antimicrobial Susceptibility Patterns of the Isolated Pathogens in Japan. J. Infect. Chemother. 26, 890–899. doi: 10.1016/j.jiac.2020.05.020 32622623

[B67] Suzuki K. Kurono Y. Ikeda K. Watanabe A. Iwamoto A. Totsuka K.. (2015). Nationwide Surveillance of 6 Otorhinolaryngological Infectious Diseases and Antimicrobial Susceptibility Pattern in the Isolated Pathogens in Japan. J. Infect. Chemother. 21, 483–491. doi: 10.1016/j.jiac.2015.03.005 26004175

[B68] Team R. C. (2019). R: A Language and Environment for Statistical Computing, Vienna, Austria (Vienna, Austria: R Foundation for Statistical Computing).

[B69] van Dongen T. M. Schilder A. G. Venekamp R. P. de Wit G. A. van der Heijden G. J. (2015). Cost-Effectiveness of Treatment of Acute Otorrhea in Children With Tympanostomy Tubes. Pediatrics 135 (5), e1182–e1189. doi: 10.1542/peds.2014-3141 25896832

[B70] Wang J. C. Pillutla P. Cordero J. Hamood A. N. (2018). Prospective Observational Case Series Evaluating Middle Ear Fluid and Tympanostomy Tubes Through Pyrosequencing. Int. J. Pediatr. Otorhinolaryngol 114, 159–165. doi: 10.1016/j.ijporl.2018.08.035 30262357

[B71] Weiss J. P. Antonelli P. J. Dirain C. O. (2019). Microbiome Analysis of Cholesteatoma by Gene Sequencing. Otol. Neurotol. 40 (9), 1186–1193. doi: 10.1097/MAO.0000000000002355 31469791

[B72] Xu J. Du Q. Shu Y. Ji J. Dai C. (2020). Bacteriological Profile of Chronic Suppurative Otitis Media and Antibiotic Susceptibility in a Tertiary Care Hospital in Shanghai, China. Ear Nose Throat J. 100 (9), NP391–NP396. doi: 10.1177/0145561320923823 32352873

[B73] Yusuf E. Halewyck S. Wybo I. Pierard D. Gordts F. (2015). Fusobacterium Necrophorum and Other Fusobacterium Spp. Isolated From Head and Neck Infections: A 10-Year Epidemiology Study in an Academic Hospital. Anaerobe 34, 120–124. doi: 10.1016/j.anaerobe.2015.05.006 25988544

[B74] Zhang Y. Xu M. Zhang J. Zeng L. Wang Y. Zheng Q. Y. (2014). Risk Factors for Chronic and Recurrent Otitis Media-a Meta-Analysis. PloS One 9 (1), e86397. doi: 10.1371/journal.pone.0086397 24466073 PMC3900534

