# Supplementary material for: Microbiota Associated With Cholesteatoma Tissue in Chronic Suppurative Otitis Media
Source: Front Cell Infect Microbiol. 2022 Apr 19;12:746428. doi: 10.3389/fcimb.2022.746428 (PMC9063009; doi:10.3389/fcimb.2022.746428)
Supplement: Supplementary file 1 [file DataSheet_1.pdf]

Table S1. Primers Used for Viral Screening

Virus	*Abbr.*	*Gene*	*Forward Primer*	*Reverse Primer*
HPV-E6	HPV	Oncoprotein E6	TCAGGACCCACAGGAGCG	CCTCACGTCGCAGTAACTGTTG
HPV-E7	HPV	Early protein E7	CCGGACAGAGCCCATTACAA	CGAATGTCTACGTGTGTGCTTTG
HPV-GP5/6	HPV	Late major capsid L1	GAAAAATAAACTGTAAATCATATT	TTTGTTACTGTGGTAGATACTAC
Adenovirus	HAdV	Hexon	GCCACGGTGGGGTTTCTAAACTT	GCCCCAGTGGTCTTACATGCACAT
Human Bocavirus NS-1	HBoV	Non-structural protein 1	TGCAGACAACGCYTAGTTGTTT	CTGTCCCGCCCAAGATACA
Rhinovirus	RV	Genome/5'UTR	TGGACAAGGTGCGAAGAG	CAAAGTAGTCGGTCCCATCC
Influenza B	InfB	Segment 4 hemagglutinin	AAATACGGTGGATTAAATAAAAGCAA	CCAGCAATAGCTCCGAAGAAA
Influenza A	InfA	Matrix proteins 1 & 2	GACCRATCCTGTCACCTCTGAC	AGGGCATTYTGGACAAAKCGTCTA
Human Metapneumovirus	hMPV	Nucleocapsid	CATATAAGCATGCTATATTAAAAGAGTCTCA	CCTATTTCTGCAGCATATTTGTAATCAG
Respiratory Syncytial Virus	RSV	cRNA/genome	GCTCTTAGCAAAGTCAAGTTRAATGATACA	GTTTTTGCACATCATAATTRGGAGT
Enterovirus	EV	Polyprotein	GGCCCCTGAATGCGGCTAATCC	GCGATTGTCACCATWAGCAGYCA
Human Coronavirus-229E	hCoV	Nucleoprotein	CAGTCAAATGGGCTGATGCA	AAAGGGCTATAAAGAGAATAAGGTATTCT
hCoV-OC43	hCoV	Nucleocapsid	CGATGAGGCTATTCCGACTAGGT	CCTTCCTGAGCCTTCAATATAGTAACC
hCoV-NL63	hCoV	Nucleocapsid	AGGACCTTAAATTCAGACAACGTTCT	GATTACGTTTGCGATTACCAAGACT
Beta-actin-2 (control)	β-actin	Exon 4	TCACCCACACTGTGCCCATCTACGA	CAGCGGAACCGCTCATTGCCAATG

Table S2. Most Prevalent Cholesteatoma-associated Bacteria (showing all taxa found in >50% of Cholesteatomas)

	Prevalence				Mean Abundance (s.d.)				
Taxon	CHOL N=25	GRAN N=34	MEMUC N=17	MEDISC N=27	CHOL	GRAN	MEMUC	MEDISC	Phylum
Bact:Porphyromonas	100	91	82	89	8.32 (12.05)	5.23 (10.00)	6.34 (10.36)	7.12 (13.67)	Bacteroidetes
Firm:Streptococcus	96	94	100	93	0.20 (0.67)	0.95 (1.61)	2.00 (4.18)	0.47 (1.00)	Firmicutes
Fuso:Fusobacterium	92	71	76	89	2.04 (3.97)	0.68 (1.35)	1.17 (2.45)	2.04 (5.09)	Fusobacteria
Firm:Peptoniphilus	92	91	88	96	7.27 (9.76)	5.84 (10.57)	5.54 (9.15)	3.05 (4.81)	Firmicutes
Prot:Gammaproteobacteria	88	91	88	96	0.60 (1.98)	6.96 (11.92)	5.69 (6.74)	4.02 (10.10)	Proteobacteria
Prot:Pseudomonas	88	94	100	81	2.30 (6.04)	2.72 (4.44)	0.84 (1.07)	1.11 (2.42)	Proteobacteria
Prot:Haemophilus	84	85	88	96	0.03 (0.08)	0.29 (0.61)	0.30 (0.67)	0.07 (0.20)	Proteobacteria
Firm:Staphylococcus	84	88	76	93	7.95 (22.15)	6.20 (17.55)	7.22 (17.28)	5.11 (13.57)	Firmicutes
Bact:Prevotella	84	76	76	81	0.98 (2.22)	1.13 (2.74)	0.97 (1.52)	2.13 (5.69)	Bacteroidetes
Acti:Propionibacterium	84	91	100	89	0.34 (1.04)	6.19 (8.94)	5.10 (5.03)	4.68 (12.26)	Actinobacteria
Acti:Corynebacterium	80	94	88	100	11.53 (26.79)	5.63 (13.77)	2.08 (2.30)	7.87 (16.28)	Actinobacteria
Prot:Alcaligenaceae	80	85	88	78	4.67 (11.75)	1.56 (3.74)	2.00 (4.94)	2.71 (6.89)	Proteobacteria
Acti:Brevibacterium	80	76	76	78	3.68 (6.81)	1.67 (4.59)	2.23 (4.34)	5.62 (12.74)	Actinobacteria
Firm:Parvimonas	80	82	82	70	3.58 (7.88)	2.46 (6.93)	1.19 (2.82)	3.24 (6.42)	Firmicutes
Bact:Bacteroides	80	79	88	78	2.23 (3.77)	2.31 (3.81)	3.17 (4.09)	3.25 (7.23)	Bacteroidetes
Prot:Enterobacteriaceae	80	85	88	89	3.28 (8.87)	2.11 (7.29)	5.29 (15.19)	4.06 (10.86)	Proteobacteria
Firm:Anaerococcus	80	62	65	74	1.04 (3.00)	0.67 (1.65)	2.27 (6.20)	0.48 (1.42)	Firmicutes
Prot:Pseudomonadales	76	79	82	74	7.16 (21.60)	8.96 (19.36)	3.67 (12.16)	5.25 (17.19)	Proteobacteria
Prot:Moraxella	72	59	65	85	0.01 (0.02)	0.17 (0.78)	0.37 (1.01)	0.12 (0.53)	Proteobacteria
Acti:Actinomyces	72	76	82	70	0.40 (1.40)	0.33 (0.55)	0.30 (0.47)	0.40 (0.84)	Actinobacteria
Firm:Bacillus	72	91	100	63	0.10 (0.33)	1.60 (2.56)	1.97 (3.01)	0.83 (2.15)	Firmicutes
Prot:Proteus	72	56	65	78	1.48 (3.42)	1.25 (3.23)	1.97 (4.40)	2.84 (7.27)	Proteobacteria
Prot:Escherichia	68	71	88	67	0.19 (0.48)	0.36 (0.69)	0.86 (1.17)	0.80 (1.90)	Proteobacteria
Firm:Finegoldia	68	68	71	70	1.64 (4.77)	1.44 (4.29)	1.90 (3.67)	1.17 (2.78)	Firmicutes
Prot:Achromobacter	64	62	59	67	3.55 (13.01)	2.75 (11.46)	0.29 (0.43)	1.45 (6.44)	Proteobacteria
Prot:Campylobacter	64	59	65	70	1.19 (2.16)	1.35 (4.23)	0.37 (0.92)	0.14 (0.34)	Proteobacteria
Firm:Family-XI-Incertae-Sedis	64	47	53	37	0.37 (0.73)	0.37 (0.92)	0.14 (0.21)	0.10 (0.28)	Firmicutes
Prot:Acinetobacter	60	65	76	63	0.28 (1.33)	0.59 (1.43)	2.46 (9.05)	0.23 (0.50)	Proteobacteria
Firm:Family-XIII-Incertae-Sedis	60	53	59	59	1.25 (3.25)	0.58 (1.40)	0.41 (0.80)	0.24 (0.41)	Firmicutes
Proteobacteria	60	62	59	63	0.01 (0.02)	0.06 (0.13)	0.15 (0.50)	0.03 (0.08)	Proteobacteria
Spir:Treponema	56	41	47	59	1.35 (2.81)	1.50 (4.04)	2.21 (4.25)	1.23 (3.24)	Spirochaetae
Acti:Corynebacteriaceae	52	68	71	59	0.19 (0.82)	0.38 (0.81)	0.22 (0.28)	0.18 (0.37)	Actinobacteria
Prot:Alcaligenes	52	32	29	59	0.41 (1.41)	1.98 (10.60)	0.08 (0.25)	3.24 (11.10)	Proteobacteria
Acti:Mobiluncus	52	44	71	48	2.12 (4.84)	2.27 (6.81)	0.88 (1.97)	0.31 (0.66)	Actinobacteria

Table S3. PERMANOVA Results Stratified by Sample-type

**A. Univariable**	**PERMANOVA P-Values**			
**Variable**	**Chol**	**Gran**	**MEDisc**	**MEMuc**
Age	0.19	**0.004**	0.34	0.20
Cholesteatoma Dx	na	**0.0005**	**0.002**	0.37
Quinolone	0.15	**0.04**	0.41	**0.008**
**B. Multivariable**	**PERMANOVA p-Values**			
**Variable**	**Chol**	**Gran**	**MEDisc**	**MEMuc**
Age	0.28	0.23	0.70	0.56
Cholesteatoma Dx	na	**0.03**	**0.002**	0.31
Quinolone	0.13	0.055	0.24	**0.02**

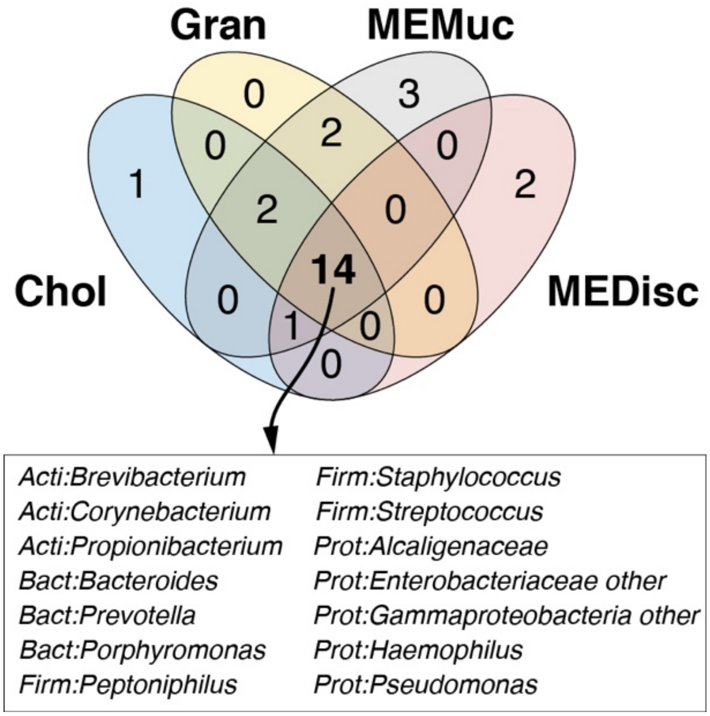

Figure S1. Overlap in highly prevalent taxa. Venn diagram shows numbers of taxa detected in ≥75% of patients for each sample-type. The 14 taxa identified in all four sample-types are listed below the diagram. Taxa names are pre-pended with phylum identifiers: Acti = Actinobacterium; Bact = Bacteroidetes; Firm = Firmicutes; Prot = Proteobacteria.

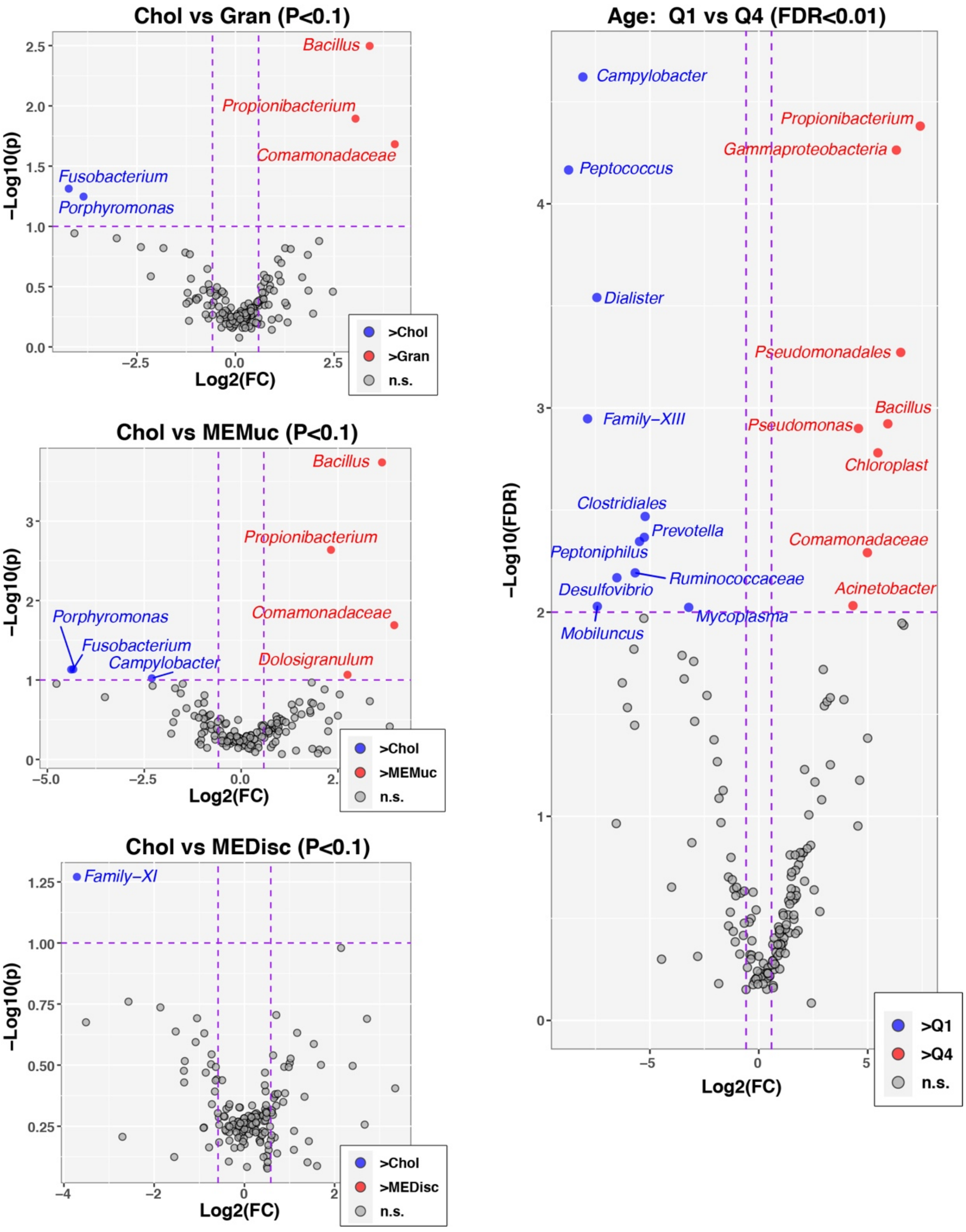

Figure S2. Individual taxa differing by sample-type or age. Between-group differences in the relative abundance of individual bacterial taxa were identified using the ANOVA-like differential expression (ALDEx2) test, which considers the compositional nature of microbiota datasets. *Vertical dashed lines* indicate fold-change cutoffs ≥1.5. *Horizontal dashed lines* show p-value cutoffs for comparisons of sample-types (Left panels; nominal p≤0.1) or age quartiles (Right panel; FDR-corrected p≤0.01). ***For sample-type comparisons,*** *blue circles in the upper left quadrants* denote taxa enriched in Chol samples while *red circles in the upper right quadrants* denote taxa enriched in the other comparison group. ***For comparison of age Q1 vs Q4,*** *blue circles in the upper left quadrants* denote taxa enriched in Q1 samples while *red circles in the upper right quadrants* denote taxa enriched in Q4 samples. n.s.=not significant

MEMuc vs. Chol Tissue

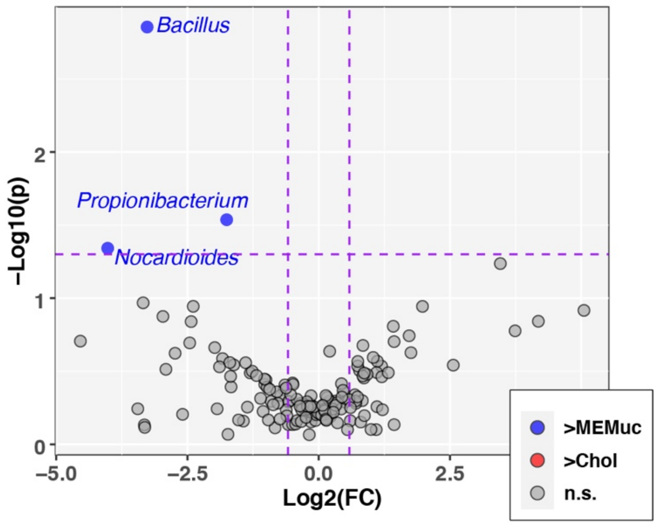

MEDisc vs. Chol Tissue

No significant taxa at cutoff of:p = 0.05

Gran vs. Chol Tissue

No significant taxa at cutoff of: p = 0.05

Figure S3. Individual taxa differing between Chol tissues and other ME sample-types among cholesteatoma patients. Between-group differences in the relative abundance of individual bacterial taxa were identified using the ANOVA-like differential expression (ALDEx2) test, which considers the compositional nature of microbiota datasets. *Vertical dashed lines* indicate fold-change cutoffs≥1.5. *Horizontal dashed lines* show p-value cutoffs for comparisons (nominal p≤0.05). *Blue circles in the upper left quadrant* denote taxa enriched in MEMuc relative to Chol while *red circles in the upper right quadrans* denote taxa enriched in Chol relative to MEMuc. No significant taxa were identified in comparing Chol to MEDisc or Gran.

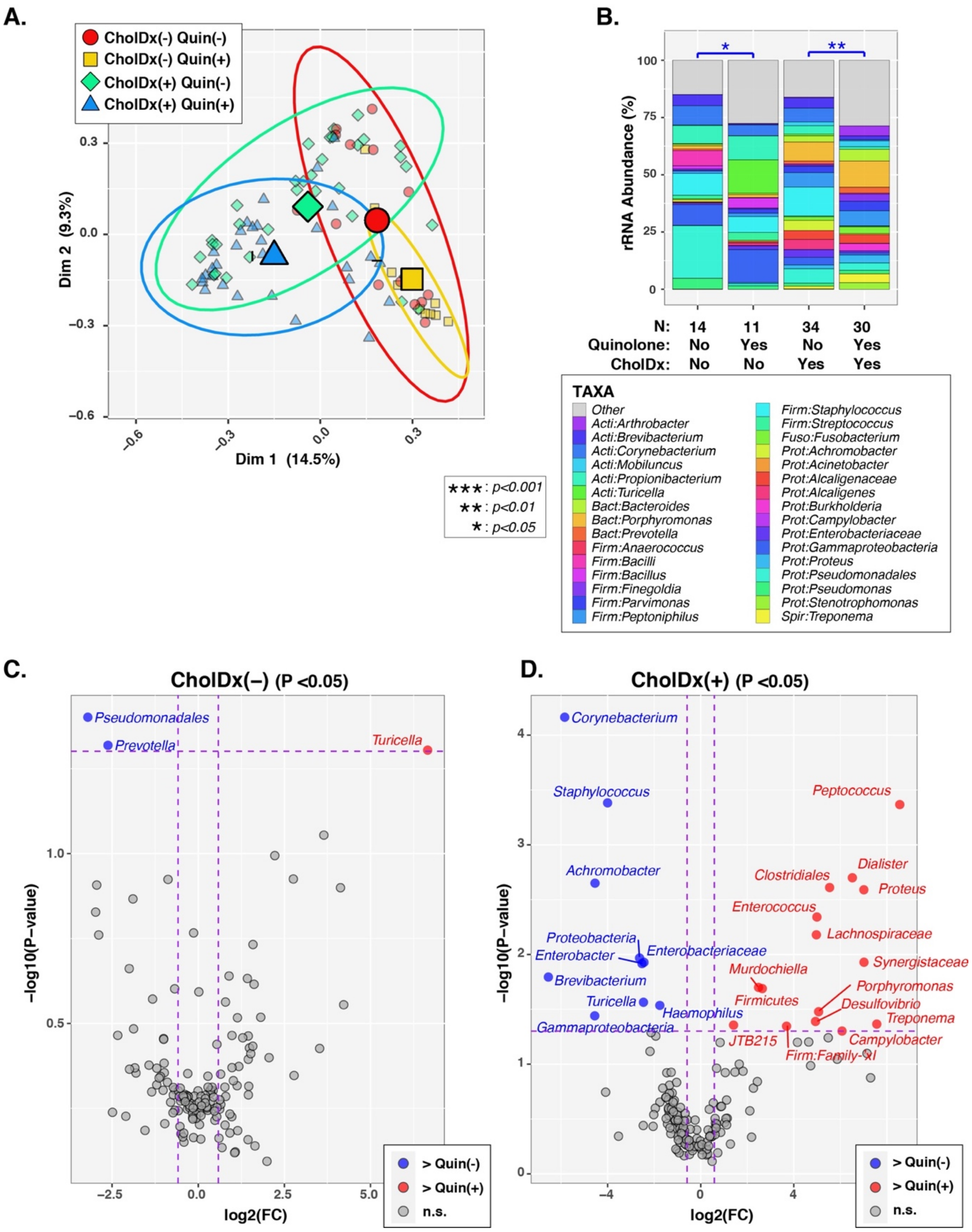

Figure S4. Effects of quinolone use on ME microbiota in subjects with and without cholesteatoma diagnosis. ***Panel A.*** Principal coordinates (PC) plots coded by cholesteatoma diagnosis [CholDx(+) vs. CholDx(-)] and quinolone use [Quin(+) vs. Quin(-)]. *Smaller symbols* designate individual subjects while *larger symbols* represent group means along both PC axes. Ellipses designate 90% confidence level for a multivariate t-distribution. ***Panel B.*** Barcharts summarizing the mean relative abundances of predominant taxa (>2%RA) in each group; rarer taxa are grouped into the "Other" category. Results of PERMANOVA tests are indicated above barcharts; tests were conducted only for 1) CholDx(-)/Quin(-) vs. CholDx(-)/Quin(+) and 2) CholDx(+)/Quin(-) vs. CholDx(+)/Quin(+) subjects. *Blue lines/symbols* indicate p-values for pairwise comparisons. ***Panel C.*** Individual taxa differing between CholDx(-)/Quin(-) and CholDx(-)/Quin(+) subjects, assessed by Aldex2 and adjusting for age and sample-type. ***Panel D.*** Individual taxa differing between CholDx(-)/Quin(-) and CholDx(-)/Quin(+) subjects, assessed by Aldex2 and adjusting for age and sample-type. *Vertical dashed lines* indicate fold-change cutoffs≥1.5. *Horizontal dashed lines* show p-value cutoffs for comparisons (nominal p≤0.05). *Blue circles in the upper left quadrants* denote taxa enriched in Quin(-) subjects while *red circles in the upper right quadrants* denote taxa enriched in Quin(+) subjects.

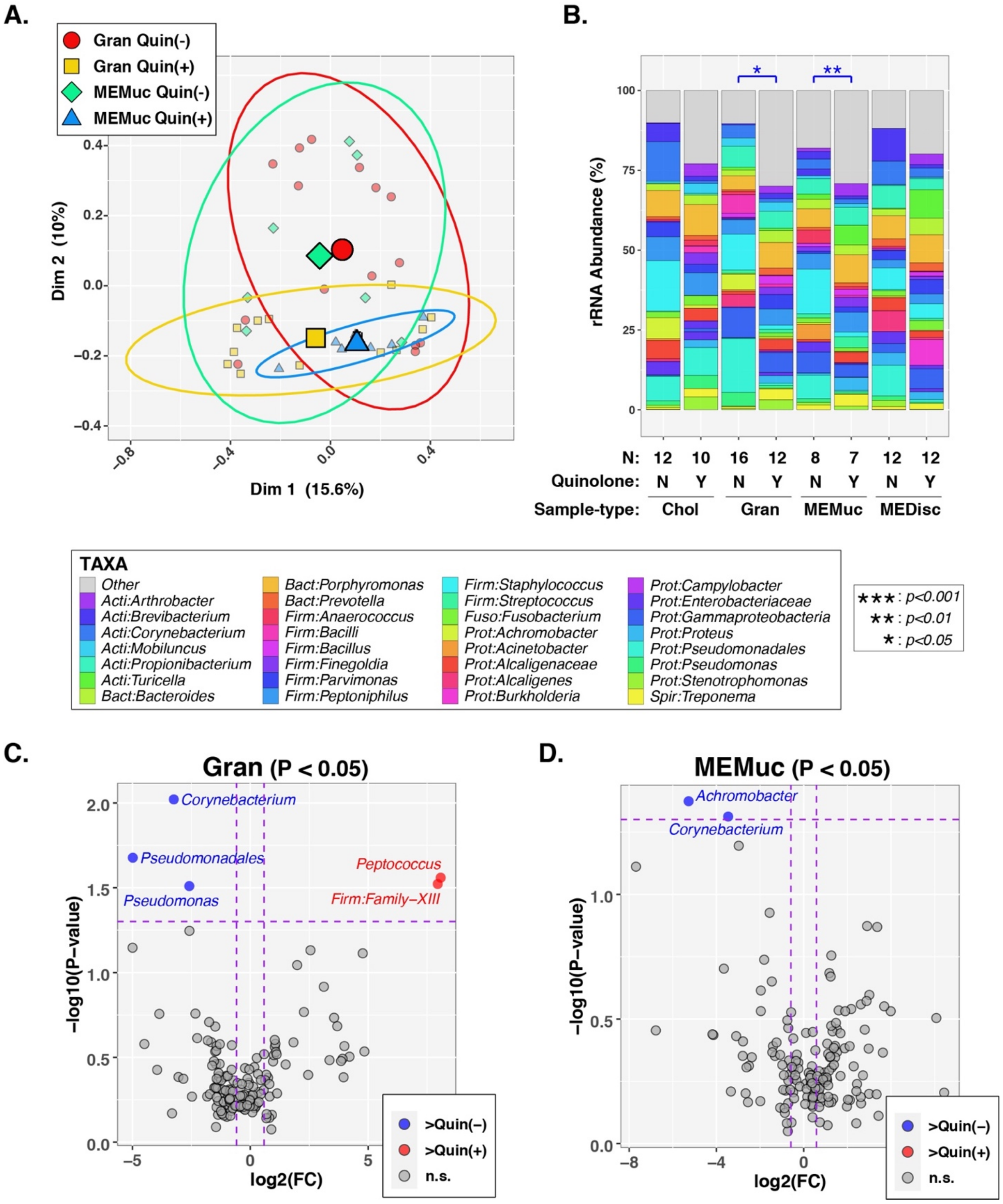

Figure S5. Effects of quinolone use on middle ear microbiota in subjects, stratified by sample-type. ***Panel A.*** Principal coordinates (PC) plots coded by sample-type (Gran vs. MEMuc) and quinolone use [Quin(+) vs. Quin(-)]. *Smaller symbols* designate individual subjects while *larger symbols* represent group means along both PC axes. *Ellipses* designate 90% confidence level for a multivariate t-distribution. ***Panel B.*** Bar charts summarizing the mean relative abundances of predominant taxa (>2%RA) in each group; rarer taxa are grouped into the "Other" category. Results of PERMANOVA tests are indicated above bar charts; tests were conducted only between Quin(+) and Quin(-) subjects within each sample-type category. *Blue lines/symbols* indicate p-values for pairwise comparisons. ***Panel C.*** Individual taxa differing between Gran/Quin(+) and Gran/Quin(-) subjects, assessed by Aldex2 and adjusting for age and cholesteatoma diagnosis. ***Panel D.*** Individual taxa differing between MEMuc/Quin(+) and MEMuc/Quin(-) subjects, assessed by Aldex2 and adjusting for age and cholesteatoma diagnosis. *Vertical dashed lines* indicate fold-change cutoffs≥1.5. *Horizontal dashed lines* show p-value cutoffs for comparisons (nominal p≤0.05). *Blue circles in the upper left quadrants* denote taxa enriched in Quin(-) subjects while *red circles in the upper right quadrants* denote taxa enriched in Quin(+) subjects.
